# Discovery of the genus *Meggoleus* Townes, 1971 (Hymenoptera, Ichneumonidae, Tersilochinae) in Peru, with the description of two new species

**DOI:** 10.3897/zookeys.163.2291

**Published:** 2012-01-09

**Authors:** Mabel Alvarado

**Affiliations:** 1 Division of Entomology, Natural History Museum, and Department of Ecology & Evolutionary Biology, 1501 Crestline Drive – Suite 140, University of Kansas, Lawrence, Kansas, 66045, USA; 2 Departamento de Entomología, Museo de Historia Natural, Av. Arenales 1256 Jesús María, Lima 14, Perú

**Keywords:** Ichneumonidae, taxonomy, new species, Peru, Neotropical region, South America

## Abstract

The genus *Meggoleus* Townes, 1971 (Ichneumonidae, Tersilochinae) currently comprises two species, one from Brazil and one from Gabon. The genus is recorded from Peru for the first time, with a range extension of the type species, *Meggoleus spirator* Townes, 1971, and the discovery of two new species – *Meggoleus fuscatus* **sp. n.** and *Meggoleus pampahermosensis* **sp. n**. A key to the world’s species is provided.

## Introduction

Tersilochinae is a cosmopolitan ichneumonid subfamily that is most species-rich in the Holarctic region (Yu et al. 2005; Khalaim 2007). Only the Palaearctic fauna has been studied moderately well, the majority of non-Palaearctic species are still undescribed (Khalaim and Sheng 2009). The Neotropical fauna of Tersilochinae is poorly known and includes only four genera, *Allophrys*, *Barycnemis*, *Meggoleus* and *Stethantyx*, and with 13 described species (Yu et al. 2005). This is the first record of Tersilochinae for Peru.

*Meggoleus* Townes, 1971 is a small, tropical genus known until now from only two species. *Meggoleus spirator* Townes, 1971, the type species, was described from Curitiba, Southern Brazil (Townes 1971), and *Meggoleus townesi* Khalaim, 2007 is known only from Gabon in equatorial Africa (Khalaim 2007). The genus was also recorded from Costa Rica but not assigned to species (Gauld 1991).

The majority of Tersilochinae are koinobiont endoparasitoids of beetle larvae, mainly Curculionidae, Chrysomelidae, and Nitidulidae (Khalaim 2011), but nothing is known of the biology of *Meggoleus*.

The aim of this paper is to describe two new species of *Meggoleus* from Peru and document a range extension for *Meggoleus spirator* to this country. A key to the world’s species is also provided.

## Materials and methods

This work is based on material of the San Marcos University Natural History Museum, Peru (MUSM). A paratype of *Meggoleus spirator* deposited in the American Entomological Institute, Gainesville, Florida (AEIC) was examined. Specimens of *Meggoleus townesi* were not examined since the original description was sufficient; this species has a distinct morphology with the epicnemial carina reaching the midline of the anterior margin of the mesopleuron and the first metasomal segment without glymmae (Khalaim 2007). I present below a key to females of the four species; males were not included because only the male of *Meggoleus townesi* is known.

Morphological terminology and the format for descriptions generally follow those of Gauld (1991) and Khalaim (2011). Photomicrographs were prepared using a Nikon D1x digital camera attached to an Infinity K-2 long-distance microscopic lens. Specimens studied herein are deposited in San Marcos Natural History Museum, Peru (MUSM) and the Division of Entomology, University of Kansas Natural History Museum (SEMC).

## Systematics

### Genus Meggoleus

Townes, 1971

http://species-id.net/wiki/Meggoleus

#### Remarks.

The genus is characterized by the labium prolonged into a tongue that is about 0.33 as long as the height of head; antenna with 15 flagellomeres; foveate groove on mesopleuron almost straight, inclined 45° from horizontal; propodeum moderately long with a narrow median longitudinal carina or basal keel between the base of the propodeum and transverse carina; fore wing vein 2*m-cu* postfurcal, pretarsal claws long, not pectinate; thyridial depression much longer than wide. The Afrotropical species, *Meggoleus townesi*, differs from the Neotropical species in that the epicnemial carina reaches the anterior margin of the mesopleuron near its midlength (in Neotropical species the epicnemial carina reaches dorsally almost to the subtegular ridge) and the first tergite lacks a glymma in *Meggoleus townesi* but is present in all known Neotropical species. However, the most striking feature of *Meggoleus* is the exceptionally large (Townes 1971; Khalaim 2007) and rounded propodeal spiracle a character not known among other ichneumonids.

### Meggoleus fuscatus

Alvarado sp. n.

urn:lsid:zoobank.org:act:3CFA76C6-DD61-45AA-851D-A81A5E9D65F4

http://species-id.net/wiki/Meggoleus_fuscatus

Figs 3 6 

#### Holotype.

♀ (Fig. 3), PERU: JU [Junín], Chanchamayo, S.N. Pampa Hermosa, 10°59'52.7"S, 75°25'34.3"W, 1757 m. 23–31.v.2011, Malaise [trap]. M. Alvarado (MUSM).

#### Paratypes.

4♀♀, same data as holotype (MUSM); 2♀♀, same data as holotype, but Pan trap (SEMC); 1♀, same locality and collector as holotype, but 75°25'35.9"W, 10°59'51.8"S, 1940 m, 23–31.v.2011, Pan trap. M. Alvarado (MUSM); 1♀, same locality and collector as holotype, but 10°59'48.9"S, 75°25'35.3"W, 1593 m, 23–31.v.2011, Pan trap (MUSM).

#### Comparison.

*Meggoleus fuscatus* can be distinguished from other Peruvian species by the long foveate groove almost reaching the epicnemial carina anteriorly (Fig. 6) and darker body coloration (Figs 3, 6).

#### Description.

♀: Body length 3.9 mm (without ovipositor); fore wing length 3.6 mm. Lateral ocellus separated from eye by ca. 2.5–2.7× ocellar diameter. Flagellum of antenna filiform, short, with 15 flagellomeres; flagellomeres elongate, first flagellomere 3–4x as long as centrally broad; penultimate flagellomere 1.6–1.7× as long as centrally broad; all flagellomeres covered by short hairs, in addition to apical long bristles. Malar space as long as basal mandibular width. Clypeus broad, usually smooth on lower part, granulate and punctate on upper part. Mandible punctate basally, upper tooth much longer than lower tooth. Face, frons, vertex and occiput finely granulate and usually finely punctate (punctures sometimes indistinct because of granulation). Temple finely and sparsely punctate, smooth between punctures; temporal orbits smooth without setae. Mesoscutum entirely granulate, indistinctly punctate; notaulus weak; mesopleuron almost smooth and punctate; epicnemial carina reaching to subalar prominence; foveate groove elongate, almost reaching to epicnemial carina, oblique, with some transverse wrinkles; metapleuron finely punctate. Propodeum with basal keel distinct, 0.75–1.0× as long as apical area; spiracle round and large, separated from pleural carina; apical area elongate, acute anteriorly, with apical longitudinal carinae reaching transverse carina anteriorly, alutaceous and coarsely punctate; dorsolateral areas usually smooth with fine, sparse punctures. Fore wing with vein 2*m-cu* unpigmented anteriorly. Tibial spurs weakly curved; pretarsal claws long, not pectinate. Metasoma with tergite I moderately slender, 3.9–4.3× as long as posteriorly broad, in dorsal view polished with a row of setae in lateral part of tergite, over lateromedian longitudinal carina, extending from base of segment to dorsad spiracle, and with some sparse setae on posterior area; tergite 2 smooth, 2.1–2.3× as long as basal broad; thyridial depression distinctly elongate, about 1.5 times as long as wide; tergites 3–6 similarly sculptured; spiracle of tergite 1 large, separation between spiracles at most 1.9–2.0× spiracle diameter (maximum diameter measured between external margins of carina round spiracle); ovipositor short, upcurved, with shallow dorsal depression near apex, without teeth.

Head black except palpi, clypeus, and mandible yellowish, and malar space, scape, and pedicel reddish. Mesosoma predominantly black, sometimes partly with reddish tinge, particularly on pronotum and mesopleuron; legs yellowish except dorsum of metafemur, mesotibia, metatibia, and meso- and metatarsomeres brown. Wing membranes hyaline and weakly infuscate; pterostigma dark brown. Metasoma with segment 1 and dorsum of tergites 2–4 dark brown; remainder of metasoma yellowish.

**Figures 1–3.**
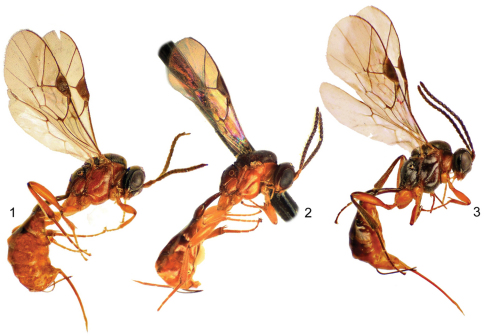
Lateral habitus of Neotropical *Meggoleus* species. **1** *Meggoleus pampahermosensis* sp. n., holotype female **2** *Meggoleus spirator* Townes, paratype female **3** *Meggoleus fuscatus* sp. n., holotype female.

#### Etymology.

The specific epithet is the Latin term *fuscatus*, meaning “darkened”, in reference to the darker body coloration of the species, compared to the other Neotropical species.

### Meggoleus pampahermosensis

Alvarado sp. n.

urn:lsid:zoobank.org:act:DA3BD80D-41E8-4710-85E6-82846B6DAEAB

http://species-id.net/wiki/Meggoleus_pampahermosensis

Figs 1 4, 8 

#### Holotype.

♀, PERU: JU [Junín], Chanchamayo, SN Pampa Hermosa, 10°59'48.9"S, 75°25'35.3"W, 1593 m, 23–31.v.2011, FIT [Flight Interception Trap], M. Alvarado (MUSM).

#### Paratypes.

3♀♀, same data as holotype (MUSM); 1♀, same data as holotype, but Pan trap (SEMC); 1♀, same data as holotype, but light trap (SEMC); 1♀, same locality and collector as holotype, 10°59'52.7"S, 75°25'34.3"W, 1757 m, 23–31.v.2011, Pan trap (MUSM).

#### Comparison.

**Figures 4–8.**
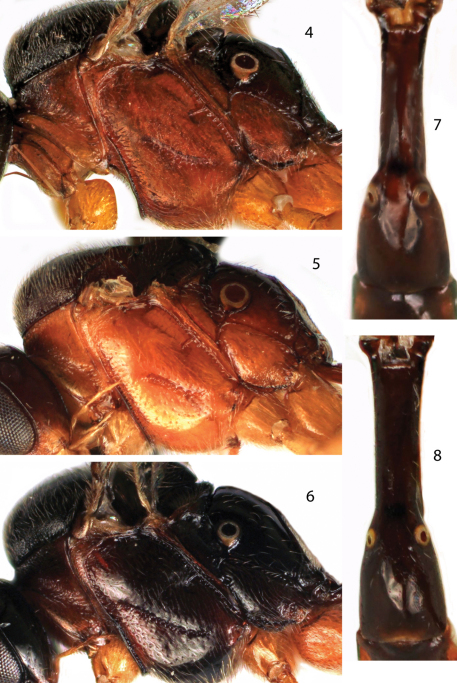
Details of Neotropical *Meggoleus* species. **4** Lateral view of mesosoma of *Meggoleus pampahermosensis* sp. n., holotype female **5** Lateral view of mesosoma of *Meggoleus spirator* Townes, paratype female **6** Lateral view of mesosoma of *Meggoleus fuscatus* sp. n., holotype female **7** Dorsal view of first metasomal tergite of *Meggoleus spirator*, female **8** Dorsal view of first metasomal tergite of *Meggoleus pampahermosensis*, holotype female.

*Meggoleus pampahermosensis* most closely resembles *Meggoleus spirator* in that the foveate groove is short, and in general body coloration (Figs 1, 4). However, the new species differs in having the first metasomal segment with the spiracles smaller, more widely spaced, and in a more lateral position (Fig. 8).

#### Description.

♀: Body length 3.6 mm (without ovipositor); fore wing length 3.2 mm. Lateral ocellus separated from eye by ca. 1.6–1.8× ocellar diameter. Fagellum of antenna filiform, short, with 15 flagellomeres; flagellomeres elongate, first flagellomere 2.3–2.7× as long as centrally broad; penultimate flagellomere 1.3–1.4× as long as centrally broad; all flagellomeres covered by short hairs, in addition to apical long bristles. Malar space 0.7–0.8× as long as basal mandibular width. Clypeus broad, usually smooth on lower part, granulate and punctate on upper part. Mandible punctate basally, upper tooth much longer than lower tooth. Face, frons, vertex and occiput finely granulate and usually finely punctate (punctures sometimes indistinct because of granulation). Temple finely and sparsely punctate, smooth between puntures; temporal orbits smooth without setae. Mesoscutum entirely granulate, indistinctly punctate; notaulus weak; mesopleuron almost smooth and punctate; epicnemial carina reaching to subalar prominence; foveate groove short, oblique, scrobiculate; metapleuron finely punctate. Propodeum with basal keel distinct, 0.7–0.8× as long as apical area; spiracle round and large, separated from pleural carina; apical area elongate, acute anteriorly, with apical longitudinal carinae reaching transverse carina anteriorly, alutaceous and coarsely punctate; dorsolateral areas usually smooth with fine, sparse punctures. Fore wing with vein 2*m-cu* unpigmented anteriorly. Tibial spurs weakly curved; pretarsal claws long, not pectinate. Metasoma with tergite 1 moderately slender, 3.1–3.2× as long as posteriorly broad, in dorsal view polished with a row of setae in lateral part of tergite, over lateromedian longitudinal carina, extending from base of segment to dorsad spiracle, and with some sparse setae on posterior area; tergite 2 smooth, 1.6–1.8× as long as basal broad; thyridial depression distinctly elongate, about 1.5× as long as wide; tergites 3–6 similarly sculptured; spiracle of tergite 1 large, separation between spiracles at most 1.8–1.9× spiracle diameter (maximum diameter measured between external margins of carina around spiracle); ovipositor short, upcurved, with shallow dorsal depression near apex, without teeth.

Head black except for palpi, clypeus, mandible, malar space, scape, and pedicel reddish. Mesosoma black except for pronotum, pleura, and sterna reddish; legs generally yellowish except base of pro- and mesotibiae, apex of metafemur, metatibia (with darker spots at base and apex), and metatarsus brown. Wing membranes generally hyaline and weakly infuscate; pterostigma dark brown. Metasoma with segment 1, dorsum of tergite 2, and basal parts of tergites 2–5 brown; remainder of metasoma yellowish.

#### Etymology.

The specific epithet is based on the type locality of Pampa Hermosa.

### Meggoleus spirator

Townes, 1971

http://species-id.net/wiki/Meggoleus_spirator

Figs 2 5, 7 

#### Material examined.

BRAZIL: 1♀ (Paratype), Campina Grande nr. [near] Curitiba, Feb. 12, 1966, H. & M. Townes (AEIC). PERU: 1♀, MD [Madre de Dios], Reserva Comunal Amarakaeri, Qda Pinquiri, 70°51'33.96"W, 12°55'29.98"S, 421 m, 03–04.vi.2011, Malaise. [trap] B. Medina y L. Huerto (MUSM).

#### Key to species of *Meggoleus* (females only)

**Table:** 

1	Epicnemial carina reaching anterior margin of mesopleuron near its midlength; first tergite without glymma (Afrotropical region)	*Meggoleus townesi* Khalaim
–	Epicnemial carina reaching subalar prominence; first tergite with glymma (Neotropical region)	2
2	Foveate groove on mesopleuron long, almost reaching to epicnemial carina (Fig. 6); metasoma brownish black to black, except pronotum reddish (Fig. 6)	*Meggoleus fuscatus* sp. n.
–	Foveate groove short (Figs 4, 5); metasoma laterally and ventrally reddish (Figs 4, 5)	3
3	Spiracle of tergite 1 large; separation between spiracles at most 1.1–1.2x spiracle diameter (maximum diameter measured between external margins of carina round spiracle), spiracles mostly located on dorsal part of tergite (Fig. 7)	*Meggoleus spirator* Townes
–	Spiracle of tergite 1 moderate sized; separation between spiracles 1.8–1.9× spiracle diameter, spiracles mostly located on lateral part of tergite (Fig. 8)	*Meggoleus pampahermosensis* sp. n.

## Supplementary Material

XML Treatment for Meggoleus

XML Treatment for Meggoleus fuscatus

XML Treatment for Meggoleus pampahermosensis

XML Treatment for Meggoleus spirator

## References

[B1] Gauld ID (1991) The Ichneumonidae of Costa Rica 1, Introduction, keys to subfamilies, and keys to the species of the lower pimpliform subfamilies: Rhyssinae, Pimplinae, Poemeniinae, Acaenitinae and Cylloceriinae. Memoirs of the American Entomological Institute 47: 1-589

[B2] Khalaim AI (2007) First records of *Meggoleus*, *Heterocola* and *Phradis* (Hymenoptera: Ichneumonidae: Tersilochinae) from the Afrotropical region, with description of four new species. African Invertebrates 48 (2): 101-110

[B3] Khalaim AI (2011) Tersilochinae of South, Southeast and East Asia, excluding Mongolia and Japan (Hymenoptera: Ichneumonidae). Zoosystematica Rossica 20 (1): 96-148

[B4] Khalaim AI Sheng M-L (2009) Review of the Tersilochinae (Hymenoptera: Ichneumonidae) of China, with description of four new species. Zookeys 14: 67-81 doi: 10.3897/zookeys.14.141

[B5] Townes HK (1971) The genera of Ichneumonidae, Part 4. Memoirs of the American Entomological Institute 17: 1-372

[B6] Yu DS Achterberg Kv Horstmann K (2005) World Ichneumonoidea 2004. In: Yu DS (Ed) Taxapad 2005 Data Base. [http://www.taxapad.com]

